# Augmented Therapeutic Potential of Glutaminase Inhibitor CB839 in Glioblastoma Stem Cells Using Gold Nanoparticle Delivery

**DOI:** 10.3390/pharmaceutics13020295

**Published:** 2021-02-23

**Authors:** Beatriz Giesen, Ann-Christin Nickel, Juri Barthel, Ulf Dietrich Kahlert, Christoph Janiak

**Affiliations:** 1 Institut für Anorganische Chemie und Strukturchemie, Heinrich-Heine-Universität Düsseldorf, 40204 Düsseldorf, Germany; beatriz.giesen@uni-duesseldorf.de; 2 Klinik für Neurochirurgie, Medizinische Fakultät, Universitätsklinikum Düsseldorf, 40225 Düsseldorf, Germany; ann-christin.nickel@med.uni-duesseldorf.de; 3 Ernst Ruska-Centrum für Mikroskopie und Spektroskopie mit Elektronen (ER-C 2), Forschungszentrum Jülich GmbH, 52425 Jülich, Germany; ju.barthel@fz-juelich.de; 4 Deutsches Konsortium für Translationale Krebsforschung (DKTK), 40225 Düsseldorf, Germany

**Keywords:** glutaminase inhibition, gold nanoparticles, drug delivery, glioblastoma stem cells

## Abstract

Gold nanoparticles (Au NPs) are studied as delivery systems to enhance the effect of the glutaminase1 inhibitor CB839, a promising drug candidate already in clinical trials for tumor treatments. Au NPs were synthesized using a bottom-up approach and covered with polymers able to bind CB839 as a Au-polymer-CB839 conjugate. The drug loading efficiency (DLE) was determined using high-performance liquid chromatography and characterization of the CB839-loaded NPs was done with various microscopic and spectroscopic methods. Despite the chemical inertness of CB839, Au NPs were efficient carriers with a DLE of up to 12%, depending on the polymer used. The therapeutic effect of CB839 with and without Au was assessed in vitro in 2D and 3D glioblastoma (GBM) cell models using different assays based on the colony formation ability of GBM stem cells (GSCs). To avoid readout disturbances from the Au metal, viability methods which do not require optical detection were hereby optimized. These showed that Au NP delivery increased the efficacy of CB839 in GSCs, compared to CB839 alone. Fluorescent microscopy proved successful NP penetration into the GSCs. With this first attempt to combine CB839 with Au nanotechnology, we hope to overcome delivery hurdles of this pharmacotherapy and increase bioavailability in target sites.

## 1. Introduction

The low survival rate of patients after diagnosis with glioblastoma (GBM), a highly aggressive brain cancer, is believed to rely on the invasive nature of the tumor, its innate recurrence and the high level of therapy resistance of the disease [1]. Moreover, the difficulty of therapeutics to sufficiently pass the blood–brain barrier (BBB) during treatment adds to the complexity of the management of patients with GBM [2,3]. A subpopulation of cells with enhanced stem characteristics (GBM stem-like cells, GSCs) have been the focus of new strategies to eradicate GBM, as they seem to be responsible for building therapy resistance due to their self-renewal properties and their ability to differentiate into a variety of cell types within the tumor [4,5,6].

In recent years, the increased glucose and glutamine uptake of tumors needed to maintain their metabolism has been exploited as a target in breast, prostate, glioma, lung, kidney, thyroid, and blood cancers [7,8,9,10,11,12,13]. Instead of converting glucose to pyruvate to produce adenosine triphosphate (ATP) like normal cells, cancer cells have been known to utilize glutamine, an amino acid contained abundantly in plasma, as a nitrogen and carbon source to synthesize ATP and nucleotides [14]. During glutaminolysis, glutamine is hydrolyzed to glutamate and ammonia by the mitochondrial enzyme glutaminase1 (GLS1), a process needed to produce substrates for the citric acid cycle and subsequently supply the cells with vital proteins and other important metabolites [15]. Thus, by inhibiting the function of glutaminases, cell apoptosis and a slower tumor growth is expected and has already been observed in a variety of cases [16].

**Figure 1:**
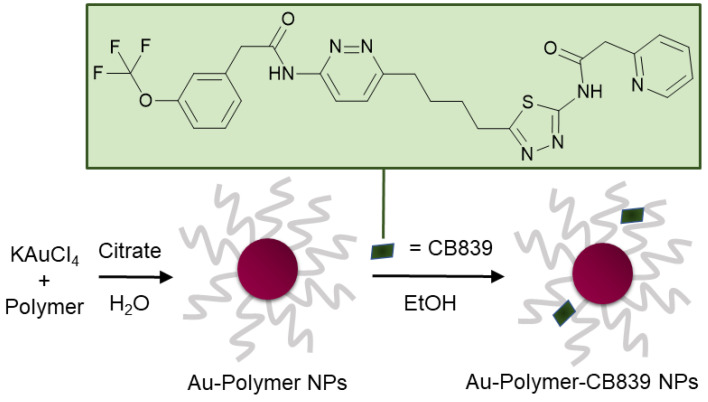
Schematic synthesis of polymer coated gold nanoparticles (Au NPs) carrying the CB839 inhibitor.

The compound *N*-[5-[4-[6-[[2-[3-(trifluoromethoxy)phenyl]-acetyl]amino]-3-pyridazinyl]butyl]-1,3,4-thiadiazol-2-yl]-2-pyridineacetamide (CB839) (Figure 1) shows great promise as a glutaminase inhibitor, with several clinical trials as cancer therapy underway [17,18]. Most recently, CB839—brand marked under the name Telaglenastat—is being investigated in a phase 2, randomized, multicenter, double-blind clinical study enrolling 120 lung cancer patients (NCT04265534). Treatment regime consists of orally administered CB839 as a supplement to food. Moreover, the tolerability of the drug in healthy adults dosing 800 mg (4 × 200 mg tablets) administered twice daily is currently being assessed in the phase 1 NCT04607512 trial.

CB839 has good oral bioavailability and a potency independent from glutamine concentration about 13 times higher than its predecessor bis-2-(5-phenylacetamido-1,3,4-thia-diazol-2-yl)ethyl sulfide (BPTES) [19]. We and others have previously shown that pharmacological GLS inhibition (GLSi) is effective to combat GSCs [20,21] and GLSi candidate CB839 is the favorable strategy to do so, as it has superior target specificity [22]. In vivo therapy studies with this drug candidate, however, have shown that—in comparison to other tissues—the brain accumulation of CB839 is significantly hindered, most likely due to the challenge of passing the BBB [19]. Moreover, this in vivo trail enforces improving the general bioavailability of CB839 into the tumor sites, as oral treatment with the compound require severely high dosages to achieve therapy efficacy. Thus, new strategies to ensure more effective penetration of this promising drug candidate into tumor cells are urgently required.

The use of gold nanoparticles (Au NPs) as therapy carrier platform surges in recent biomedical applications due to their straight-forward synthesis and various, modifiable intrinsic functional properties [23]. Furthermore, nanoparticles present an enhanced permeation and retention (EPR) effect in tumors and can successfully transverse the cell membrane barrier of GSCs, as previously demonstrated [24,25]. In addition, Au NPs as pharmacological carriers can be beneficial to overcome the obstacles of administrating poorly water-soluble compounds such as CB839, thereby reducing the required doses to be administered and possibly minimizing risks of side-effects in patients.

Unlike prior glutaminase inhibitors, new generation inhibitors such as BPTES or BPTES analogues like CB839 do not contain highly reactive functional groups in their structure [26]. While this is considered a biological advantage because the drug cannot form toxic intermediates with proteins, it complicates a possible binding to the surface of a NP. Because no covalent bonds can be easily formed between CB839 and NP capping ligands, the use of conventional drug-attachment methods such as *click*-reactions, 1-ethyl-3-(3-dimethylaminopropyl) carbodiimide hydrochloride/N-hydroxy succinimide (EDC/NHS) chemistry and cross-linkers (as seen for doxorubicin or anticancer taxanes such as paclitaxel or docetaxel) [27,28,29] is not possible without significant structure modification. Since such an alteration poses a high risk to influence the promising clinical effect known for this inhibitor, choosing a polymer as a surface ligand can provide the necessary matrix to load the CB839 molecules to the NPs. Through physical adsorption, polymers are capable of easily embedding drugs and macromolecules without the need for chemical modification, sometimes even achieving a higher loading than encapsulation techniques [30]. Moreover, polymers can increase the retention time of drugs, as well as their circulation in blood and are able to release them when the conjugate is exposed to endo- and exogeneous tumor stimuli [31].

In this work, a variety of biocompatible polymers were extensively examined regarding their ability to load CB839 onto the surface of the polymer-coated Au NPs (Figure S1, Supplementary Materials). After a fast preparation of Au-Polymer-CB839 NPs without the need of additional linkers (Figure 1), the efficacy of the CB839 inhibitor with and without the Au carriers was assessed in vitro using GBM neurospheres. These neurospheres are capable of reflecting the original tumor environment more closely due to their three-dimensional structure, compared to classical disease models using monolayer culturing technology [32]. For example, a hypoxic micro-environment could be formed in spheres from the outside to the inner core or the drug uptake can be slower for the inner cells than for the outlaying cells [33]. Since the low toxicity of the individual components of the conjugate (Au NPs, polymers and CB839 on non-tumor cells) is well known [22,34,35,36], we focused on the synthesis of novel Au-Polymer-CB839 NPs and the assessment of its therapeutic potential compared to the neat counterpart. Our results advocate Au NPs to effectively improve the therapeutic potential of anti-tumor metabolic therapies.

To our knowledge, CB839 has only been combined with purely polymeric NPs and liposomes to facilitate its administration [37,38] but this is the first time it is attached to Au NPs. Contrary to commonly used assays such as MTT, Cell-Titer-Glo^®^ or Annexin A5, our focus lied further in the optimization of methods to assess the viability of the GSCs after treatment with Au-Polymer-CB839 NPs that do not rely on optical detection or addition of reagents. Au NPs may disturb assay readouts through their ability to absorb visible light, lumines, quench fluorescent signals or simply by interacting with assay components [39,40,41]. Thus, it is of high importance to have reliable and easy-to-apply methods to test the toxicity of drugs when using Au NPs.

## 2. Materials and Methods

### 2.1. Materials

Potassium tetrachloridoaurate (III), polyvinyl alcohol (PVA) Mowiol 4-88, *M_w_* ~31,000 g/mol, poly(ethylene glycol) methyl ether thiol (ThioPEG), *M_w_* ~800 g/mol, branched polyethylene imine (PEI) *M_w_* ~25,000 g/mol, polyvinyl pyrrolidone (PVP), *M_w_* ~3500 g/mol, fluorescein isothiocyanate (FITC), crystal violet, 4-nitro blue tetrazolium chloride, 4′,6-diamidino-2-phenyl-indol-dihydro-chloride (DAPI), Trypan Blue and Heparin (#H0878) were purchased from Sigma Aldrich, Darmstadt, Germany. Sodium citrate dihydrate was from J.T. Baker Chemicals, Schwerte, Germany and dimethyl sulfoxide (DMSO, 99.7% purity) from Honeywell, Offenbach, Germany. Telaglenastat (CB839) was purchased from Adooq Bioscience, Irvine, CA, USA. Ethanol and methanol (Merck, Darmstadt, Germany) were of p.a. purity. Penicillin/Streptomycin (#P4333) was also from Merck, Darmstadt, Germany. Phosphate-buffered saline (PBS, Gibco #10010015), Dulbecco’s Modified Eagle Medium without pyruvate (DMEM, Gibco #11965092), B27 supplement (Gibco #17504044), Ham’s F12 Nutrient Mix (Gibco #11765047), Antibiotic-Antimycotic (Gibco #15240096), fetal bovine serum (FBS, Gibco #11965092), poly-d-lysine (Gibco #A3890401), agarose (Gibco #18300012) and slide mounting solution (eBioscience™ Fluoromount-G™, #00-4958-02) were from Thermo Fisher Scientific, Schwerte, Germany. Human epidermal growth factor (#AF-100-15) and human basic fibroblast growth factor (#AF-100-18B) were from Peprotech, Rocky Hill, NJ, USA and the tissue medium (#1620C) from KliniPath, Duiven, Netherlands. The Spectra/Por^®^ dialysis membrane was purchased from Spectrum Laboratories, Schwerte, Germany. All materials were used without further purification. The Milli-Q^®^ purification system was used to treat water for all reactions.

### 2.2. Methods

*Transmission electron microscopy (TEM)*: 10 µL of each gold solution (in water for AuCit, AuThioPEG, AuPVP, AuPVA, and AuPEI; in ethanol for AuR-CB839 NPs) were dropped without dilution onto a 200 μm carbon-coated copper grid (from Electron Microscopy Sciences #CF200-CU, Munich, Germany), followed by drying in air. An FEI Tecnai G2 F20 electron microscope [42] operated at 200 kV accelerating voltage was used and TEM images were recorded with a Gatan UltraScan 1000P camera. Image calibration was done using Debye-Scherrer patterns recorded from a gold reference sample (S106, Plano GmbH, Wetzlar, Germany). To determine the NP size and size distribution, over 100 particles were counted using the Gatan Digital Micrograph software.

*Scanning transmission electron microscopy (STEM)* measurements were carried out using a high-angle annular dark-field (HAADF) detector combined with energy-dispersive X-ray (EDX) mapping. The sample preparation was the same as for TEM.

*Fourier transform infrared (IR)* spectra were recorded on a Bruker TENSOR 37 spectrometer in attenuated total reflection mode (Platinum ATR-QL, Diamond) between 550–4000 cm^−1^ after solvent removal from purified gold samples.

*Ultraviolet–visible (UV-VIS)* spectra were measured on a UV-2450 spectrometer (Shimadzu, Kyoto, Japan) using gold solutions and further analysis was done using the UVProbe software (Shimadzu).

*Fluorescence spectroscopy*: Gold solutions were diluted 1:10 in PBS, placed in quartz glass cuvettes and analyzed with an excitation of 490 nm on a FluoroMax-4 Spectrofluorometer from HORIBA Scientific, Irvine, CA, USA.

*Thermogravimetric analysis* (TGA) was carried out after solvent removal from purified gold samples on a Netzsch TG209 F3 Tarsus (Netzsch, Selb, Germany) under nitrogen atmosphere between 30 and 1000 °C at a heating rate of 5 K min^−1^.

*Dynamic light scattering (DLS):* Hydrodynamic diameters were determined with a Malvern Nano S Zetasizer instrument with a HeNe laser at a wavelength of 633 nm by diluting the ethanolic AuPVA-CB839 sample 1:20 in H_2_O or DMEM.

*High-performance liquid chromatography* (HPLC): Detection and quantification of CB839 in the supernatant samples was done using a Shimadzu LC 20AT prominence instrument with an SPD-M20A detector and a Luna C18(2) (250 × 4.60 mm, 5 micron) column from Phenomenex^®^. The sample loop volume was 20 µL and the absorbance was detected at a wavelength of 254 nm. The mobile phase consisted of 33% methanol and 67% H_2_O with a flow rate of 1 mL/min. Before each measurement, the column was flushed with the MeOH/H_2_O mixture for 30 min. The total run time per measurement was 13 min, whereas the CB839 peak could be found after approximately 9 min. Quantification of CB839 was done via peak integration after making a standard calibration curve.

### 2.3. Nanoparticle Synthesis

*Citrate-coated gold nanoparticles (AuCit)*: 20 mg (53 µmol) of KAuCl_4_ were dissolved in 200 mL of H_2_O and the solution was heated to 100 °C while stirring at 250 rpm. After addition of 93 mg (319 µmol) of sodium citrate, the color changed to bright red. The AuCit NP suspension was washed with 200 mL of H_2_O using centrifugation (22,000 rcf, 1 h, 4 °C) and resuspended in 200 mL of water before reaction with PEG and PVP or in ethanol before loading with CB839.

*ThioPEG-coated gold nanoparticles (AuThioPEG) and PVP-coated gold nanoparticles (AuPVP)*: The aqueous AuCit NP solution (200 mL) was reacted with ThioPEG or PVP in excess (100 µmol) and stirred at room temperature for 24 h. After separation via centrifugation, the polymer-coated Au NPs AuThioPEG and AuPVP were resuspended in 200 mL of ethanol.

*PVA-coated gold nanoparticles (AuPVA)*: 20 mg (53 µmol) of KAuCl_4_ were added to a PVA solution (33 mg in 200 mL of H_2_O) and heated to 90 °C. When the temperature was reached, 29 mg (100 µmol) of sodium citrate were added and the solution was continued to stir for 30 min until it turned dark red. After washing with 200 mL of water and centrifuging, the AuPVA NPs were resuspended in 200 mL of ethanol.

*PEI-coated gold nanoparticles (AuPEI)* were synthesized as described previously [25].

### 2.4. Loading of CB839 to Gold Nanoparticles (AuR-CB839, R: Cit, ThioPEG, PVP, PVA, PEI)

50 mL of the ethanolic solutions of AuCit, AuThioPEG, AuPVP, AuPVA and AuPEI NPs were combined with a 1 g/L CB839 stock in ethanol to obtain a final drug concentration of 0.05 g/L. The solutions were heated to 40 °C for 30 min and stirred for another 72 h at room temperature. After centrifugation for 1 h at 22,000 rcf and 4 °C, the supernatants were collected and used to quantify the unloaded amount of the drug which remained in solution via HPLC. Using the HPLC values, the mass of CB839 in the NPs was calculated by subtracting the CB839 amount used from the amount found in the supernatants. For each batch, the AuR-CB839 NPs pellets were resuspended with a specific volume of DMEM so that in each AuR-CB839 NP dispersion the concentration of CB839 was equivalent to 1 µmol/L. Since each NP type loaded different amounts of CB839 (see Section 3.2), the DMEM volume was adjusted depending on the sample. This procedure was repeated for each 50 mL NP batch synthesized, whereas HPLC results varied by about 1–6% between batches.

### 2.5. Synthesis of Fluorescent PVA Gold Nanoparticles (AuPVA-FITC)

FITC conjugated AuPVA nanoparticles were synthesized according to a two-step process based on our previously published reaction method [25]. In short, FITC was reacted with the pre-formed AuPVA NPs (synthesized as explained in Section 2.3). To increase the solubility of FITC, 50 mg (0.13 mmol) of the solid were dissolved in DMSO to form a FITC DMSO solution with a concentration of 4 g/L. This DMSO solution was then diluted to 0.05 g/L in water (FITC stock solution). Five milliliters of this stock solution were added to 50 mL of the aqueous AuPVA NP suspension, stirred at room temperature for 8 h and dialyzed through a 3.5 kDa Spectra/Por^®^ membrane against PBS for 24 h. AuPVA-FITC NPs were washed with 50 mL of H_2_O and redispersed in 50 mL of DMEM.

### 2.6. Cell Cultures

JHH520 cells were provided by G. Riggins (Baltimore, MD, USA), GBM1 by A. Vescovi (Milan, Italy) and BTSC407 by M.S. Carro (Freiburg, Germany). GBM neurospheres were cultured in DMEM without pyruvate, 2% B27 supplement, 30% Ham’s F12 Nutrient Mix, 20 ng/mL human epidermal growth factor, 20 ng/mL human basic fibroblast growth factor, 5 µg/mL heparin and antibiotic-antimycotic solution. U87 cells, kindly provided by A. Weyerbrock (University Freiburg, Freiburg, Germany), were cultured adherently in DMEM medium supplemented with 10% FBS. All cells were cultured in the presence of 1% Penicillin/Streptomycin. The absence of mycoplasma contamination was tested for all cells and their genetic identity was validated using short tandem repeat analysis as previously published [43]. Ethical approval for the use of the cell models to study brain cancer biology was from the ethical commission of the medical faculty of Heinrich-Heine University (study ID 5841R, initial approval 31March 2017, revised and renewed 16 September 2019). For all the functional assays we applied the following treatment conditions: Volume adjusted media treatment (control); 1 µmol/L CB839 or Au NP suspension containing 1 µmol/L CB839. CB839 was dissolved in ethanol and for a 3 mL well, 1.7 µL of this stock solution was added. We chose 1 µmol/L as our standard in vitro substance treatment condition as this concentration is generally considered sufficient to achieve a corresponding peak serum level in clinical testing [44]. Moreover, our group previously showed that this concentration of CB839 is a suitable parameter setting to conduct meaningful assays using the same preclinical models [22].

### 2.7. Colony Formation Assays

*Poly-d-Lysine method*: A solution of 0.1 g/L of poly-d-lysine was diluted with sterile PBS to a concentration of 50 µg/mL and used to coat six-well flat-bottom plates. After 1 h of incubation at room temperature, the solution was removed and the plates were washed 3-times with sterile H_2_O and left to dry on a sterile bench for 2 h. Subsequently, 500 suspension cells (GSCs) were seeded successfully attaching completely within 24 h. After that, the culture medium was replaced with fresh medium containing CB839 or AuR-CB839 NPs in DMEM with a final drug concentration of 1 µmol/L per well. After a 72 h incubation period at 37 °C, the NP-medium was removed and new culture medium was added every 3 days for 3 weeks. Finally, the plates were washed with PBS and the colonies were fixed using ice-cold methanol. Prior to counting the colonies, these were stained with a 0.5 vol.% crystal violet solution in methanol, washed with water and air-dried. Due to the nature of intrinsic adherent growth of U87 cells, these plates were not coated with poly-d-lysine.

*Agarose method* [45]: Prior to embedding the cells into the soft-agar-medium dilution, the cells were incubated with CB839 or AuR-CB839 NPs in DMEM (1 µmol/L) for 72 h. Six-well flat-bottom plates were coated with 1.5 mL of neurosphere medium containing 1 vol.% of melted agarose at 70 °C for 1 h. 2 mL of the treated cell suspension (1000 cells/well) with 0.6 vol.% agarose were added and incubated for 1 h at room temperature before adding 2 mL medium on top. After this time, the medium on the top layer was replaced with 2 mL fresh medium every 3 days for 3 weeks. At last, the top layer was removed and the plates were incubated overnight at 37 °C with 1 mL of a 1 g/L 4-nitro blue tetrazolium chloride solution in PBS before counting the formed colonies.

All experiments with both methods were done in triplicates and the results were expressed as mean values accompanied by standard deviations.

### 2.8. Fluorescent Microscopy

After synthesis, AuPVA-FITC NPs were dialyzed, centrifuged and resuspended in 50 mL of DMEM (see Section 2.5). In Equation (4) (Section 3.2), we estimate the number of AuPVA NPs per batch to 5 × 10^17^, thus giving a concentration of 5 × 10^17^ NPs/50 mL or 10^19^ NPs/L. From the Au-FITC-DMEM dispersion, 5 µL were added to a 1 mL-well containing 100,000 GBM cells. Subsequently, we estimate the concentration of AuPVA-FITC NPs to be 5 × 10^13^ NPs/mL (or the ratio 5 × 10^8^ NPs/cell), based on the amount of gold precursor used for each synthesis batch and assuming a 100% conversion (cf. Equation (4)). GBM cells were grown in spheroid culture (70–150 µm) and incubated with AuPVA-FITC NPs for 1 h. At the end of the incubation, the spheroids were washed three times with PBS and fixed with 70% methanol for 15 minutes. Then the fixed spheres were processed for frozen sections. Briefly, the spheres were embedded into frozen tissue medium and frozen/stored at −20 °C until they were processed for sectioning. The specimens were cut into 5–7 µm sections using a CM1900 Cryostat (Leica, Nussloch, Germany). For the staining process, the sections were rinsed 3-times with PBS and incubated with a DAPI solution (0.1 μg/mL in PBS) for 2 min. After rinsing the slides with PBS, the specimens were either directly mounted in slide mounting solution and covered with a coverslip or were further treated with Trypan Blue to quench extracellular fluorescence. A 0.1 vol.% Trypan Blue solution was added to the section, followed by direct mounting and covering the sample. The slides were analyzed using a Zeiss Axiovision Apotome.2 confocal microscope (Zeiss, Oberkochen, Germany) and the software ZEN Blue (2.3, SP1, black, 64 bit, release version 14.0.0.0 also from Zeiss).

## 3. Results and Discussion

### 3.1. Synthesis and Characterization of Au NPs

In order to bind CB839 to a nanocarrier, several polymeric capping ligands with different reactive groups were deposited on the surface of Au NPs as anchors. The synthesis of these gold “nanovehicles” consisted mainly of a reduction of an equal amount of gold(III) salt KAuCl_4_, using sodium citrate as a reducing agent. Starting from citrate coated gold (AuCit) NPs, made with a modification of the *Turkevich* method [46], spherical monodisperse particles were synthesized (vide infra), which were later used in a ligand exchange reaction to obtain polymer coated NPs, e.g., with poly(ethylene glycol) methyl ether thiol (AuThioPEG NPs) and with polyvinyl pyrrolidone (AuPVP NPs), respectively. Polyvinyl alcohol-coated gold (AuPVA) NPs were synthesized in a straight-forward *one-pot* reaction. The polymers ThioPEG, PVA, PVP, and PEI were chosen due to their known biocompatibility and their affinity for gold, as well as to provide a variety of different functional groups, which might successfully load CB839 onto the nanocarriers. Additionally, AuCit NPs were also included to see if Au presents any interaction with CB839 before any ligand exchange reaction with polymers.

**Figure 2:**
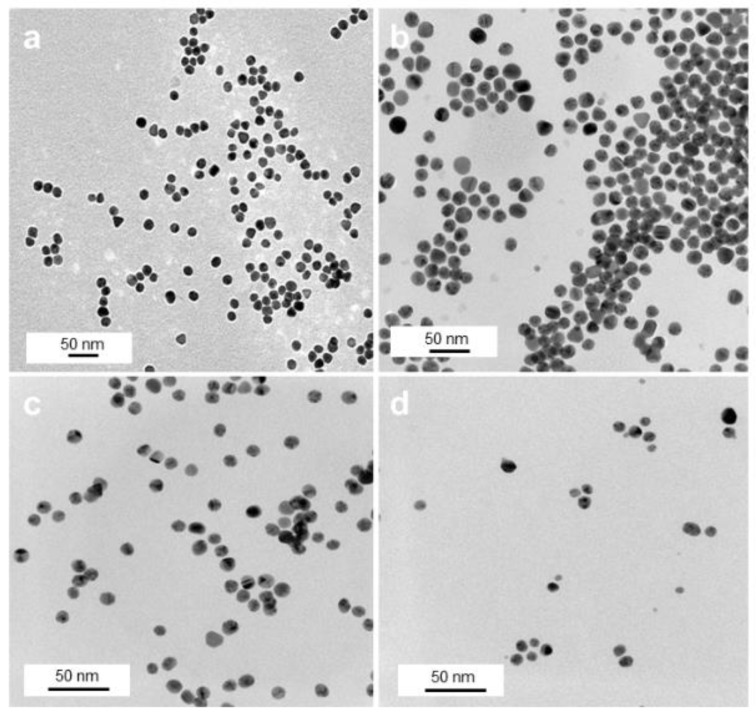
TEM images of AuCit (**a**), AuThioPEG (**b**), AuPVP (**c**), and AuPVA (**d**) NPs, captured at 50,000× (**a**), 100,000× (**b**), and 150,000× (**c**,**d**) magnification.

TEM investigations of these samples show an overall spherical morphology of the Au NPs with almost no degree of agglomeration (Figure 2) and NP sizes between 8 and 15 nm (Figure S2, Supplementary Materials (SM)). While citrate NPs had a size of 15 ± 2 nm (Figure 2a), a subsequent citrate-to-polymer exchange with ThioPEG resulted in NPs with an unchanged size of 15 ± 2 nm (Figure 2b) and with PVP gave NPs with 11 ± 2 nm average size (Figure 2c). By using the same amounts of gold precursor in the presence of PVA, spherical AuPVA NPs with a size of 8 ± 2 nm (Figure 2d) and with PEI smaller-sized 4 nm AuPEI NPs [25] were synthesized and evaluated as potential drug delivery systems.

After reacting the various Au-Polymer NPs with CB839, no apparent size increase was observed for any of the NP types except for AuPEI (Figure S3, SM). There was however a slight reorganization between the Au cores, especially in the case of AuPVA and AuPVP NPs (Figure S3c,d, SM), most likely due to an alteration in surface chemistry and charge after interaction of the polymers with CB839.

### 3.2. Quantification of CB839 Loading

Subsequently, the amount of CB839 attached to the NPs was examined by collecting supernatants of all samples after centrifugation and quantifying the not adsorbed, remaining drug concentration via HPLC analysis. For this purpose, a method capable of efficiently separating CB839 in all NP samples was developed and optimized. By using a mixture of methanol and water as mobile phase, CB839 could be identified after a retention time of only 9 min and its concentration could be calculated with a standard calibration curve (Figure S4, SM). The drug loading efficiency (DLE) of AuCit, AuThioPEG, AuPVA, AuPVP and AuPEI NPs was calculated according to Equation (1) and results are displayed in Table 1.

(1) $$\mathrm{Drug}\;\mathrm{Loading}\;\mathrm{Efficiency}\;(\%)\;=\;\frac{Total\;amount\;of\;CB839\;used-remaining\;CB839\;amount\;in\;supernatant}{Total\;amount\;of\;CB839\;used}\times 100\%\;=\;\frac{Amount\;of\;loaded\;CB839}{Total\;amount\;of\;CB839\;used}\times 100\%$$

From all five ligands used to cover the gold NPs (Figure S1, SM), PVA seems to be the most effective in loading up to 12% CB839 on the surface of NPs. This finding can probably be explained by the strong interaction of the amide group in CB839 with the numerous hydroxyl groups in the PVA chains, which has been previously exploited to bind or stabilize hormones, proteins and other pharmaceuticals [47,48]. Here, the hypothesis is that the OH groups of PVA can act both as hydrogen bond acceptors from the amide-NH groups of CB839 and donors to the aromatic nitrogen atoms and to the amide C=O groups of CB839, which leads to stronger supramolecular interactions with CB839, compared to the other polymers. Similarly, the different properties between NP types such as core size, polymeric shell and surface charge can affect the loading efficiency of each Au NP. The small size and positive charge of AuPEI NPs [25] seem to be counterproductive to load CB839. Larger 15 nm AuCit NPs show the second highest DLE after AuPVA NPs, due to possible electrostatic interactions with CB839. Aside from NP size and surface charge, the chemical nature of the polymer shell used for Au NPs can also play an important role on the DLE. Citrate and PVA feature strong hydrogen bond donor and acceptor OH groups. The NH and NH_2_ groups in PEI are already weaker H bond donors and acceptors, with the latter getting lost upon protonation. Also, upon protonation, the H bond donor function of the NH_2_^+^ and NH_3_^+^ groups will then be engaged by the counter anion. PVP is no H donor but only a week acceptor, while ThioPEG has no H bonding capabilities. Even though in theory a stronger CB839 binding capability was expected from PVP due its hydrogen bond accepting C=O groups, AuThioPEG showed a higher DLE (Table 1). A reason for this counterintuitive behavior might have to do with the polymer chain arrangement around the Au NPs. ThioPEG with *M_w_* ~800 g/mol has shorter chain lengths than polyvinyl pyrrolidone (PVP) with *M_w_* ~3500 g/mol. Shorter polymer chains cannot form a dense polymer layer as longer ones can. Thus, shorter polymer chains will be more amenable to embed guest molecules.

Even though DLE results give an overall impression of the loading success among samples, they are dependent of the concentration of CB839 used and thus only describe the efficiency of each specific reaction. For better interpretation of the experiments and the later application of the CB839-NPs, calculating the amount of CB839 molecules that are loaded per NP might be more relevant. This was done taking AuPVA-CB839 NPs as an example.

Based on the spherical shape and the radius of Au NPs (e.g., *r* = 4 nm) determined by TEM measurements, the volume of a single NP can be calculated by Equation (2):

(2) $$V_{NP}=\frac{4\;}{3}\times \;\pi \times \;r^{3}=268\;{\mathrm{nm}}^{3}$$

and its mass with Equation (3) (density of Au, ρ_Au_ = 19.32 g/cm^3^):

(3) $$m_{NP}=\rho \times V=5.18\times \;10^{-18}\;g.$$

Since 5 mg KAuCl_4_ were used in the reaction, the mass of Au in the salt (*m_Au_* = 2.6 mg), assuming a 100% conversion from KAuCl_4_ to Au NPs, can be used to determine the total number of Au NPs (*N_NPs_*) in the sample:

(4) $$N_{NPs}=\frac{m_{Au}}{m_{NP}}=5\;\times \;10^{17}.$$

From the molar amount of CB839 applied in the synthesis of AuPVA-CB839 NPs, the number of CB839 molecules can be calculated as:

(5) $$N_{CB839}=n_{CB839}\times N_{A}=2.6\times 10^{18}$$

*n_CB_*_839_ = 4.4 × 10^–6^ mol, *N_A_* = 6.022 × 10^23^ mol^−1^ (Avogadro constant).

Since the AuPVA NPs loaded 12% of the added CB839, according to the DLE results, the number of CB839 molecules loaded onto the NPs (*N_CB839loaded_*) is:

(6) $$N_{CB839loaded}=N_{CB839}\times 0.12=3.2\times 10^{17}\;molecules.$$

Finally, the number CB839 molecules loaded per NP (“Loading yield”) can be calculated using Equation (7):

(7) $$\mathrm{Loading}\;\mathrm{yield}=\frac{N_{CB839loaded}}{N_{NP}}=0.6.$$

Thus, we conclude that approximately 60% of AuPVA NPs carry one drug molecule. Since this estimation was made based on the ideal 100% NP formation from the gold precursor, this value represents the lowest possible loading of CB839 onto AuPVA NPs and might be higher in reality.

**Figure 3:**
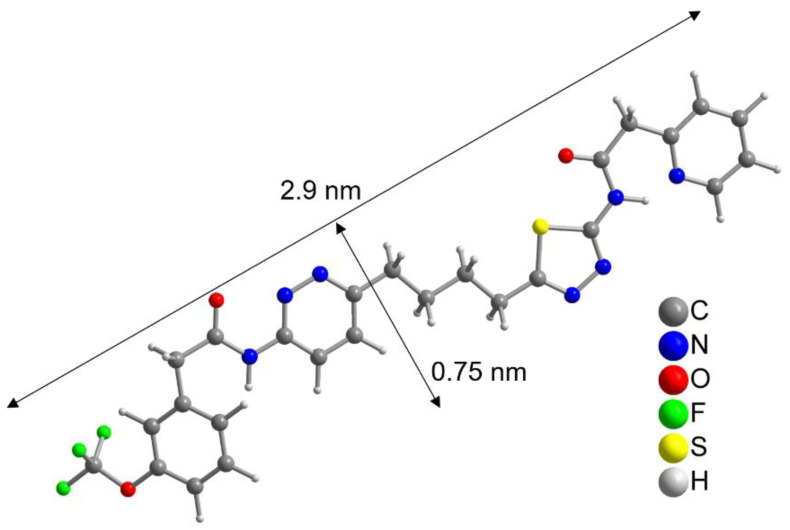
Linearized structure of the CB839 molecule. For the folded conformations in the glutaminase C complex, see Figure S5, SM.

To further evaluate this result, the size of the CB839 molecule was compared to the size of a NP. With help of a crystal structure published by Huang et al. [49], we estimated the length of the CB839 molecule in its folded conformations to be approximately 2 × 0.8 nm, and in its linear form 2.9 × 0.75 nm, taking the van der Waals radii into account (Figure 3 and Figure S5, SM). Therefore, since the size of CB839 is in the same order of magnitude as the size of a NP, it would be difficult to achieve a much higher CB839 loading with NPs of this small size. Even though we believe 60% to be a good result, improvement of the loaded amount might be possible by adjusting reaction parameters or using larger NP sizes. While these DLE values are the result of experiments using a single CB839 molar amount of *n_CB_*_839_= 4.4 × 10^–6^ mol, we will continue trying to improve the DLE of AuPVA NPs in the future by testing additional CB839 concentrations in the reaction.

### 3.3. Physicochemical Characterization of AuPVA-CB839 NPs

Prior to its application in cells, the physicochemical properties AuPVA-CB838 NPs, the NP type with highest CB839 loading, were investigated in-depth. Besides quantifying the amount of CB839 bound to the NPs, the immobilization of the drug on the surface of the AuPVA NPs was confirmed through various spectroscopic methods. IR spectroscopy of the dried NP-drug conjugate showed characteristic C-F stretching (1142 cm^−1^) and bending (735 cm^−1^) vibrations of the CF_3_ group of CB839, as well as the N-H band of its amide group (1534 cm^−1^) and the aromatic C-H bending (703 cm^−1^) vibration (Figure S6, SM).

**Figure 4:**
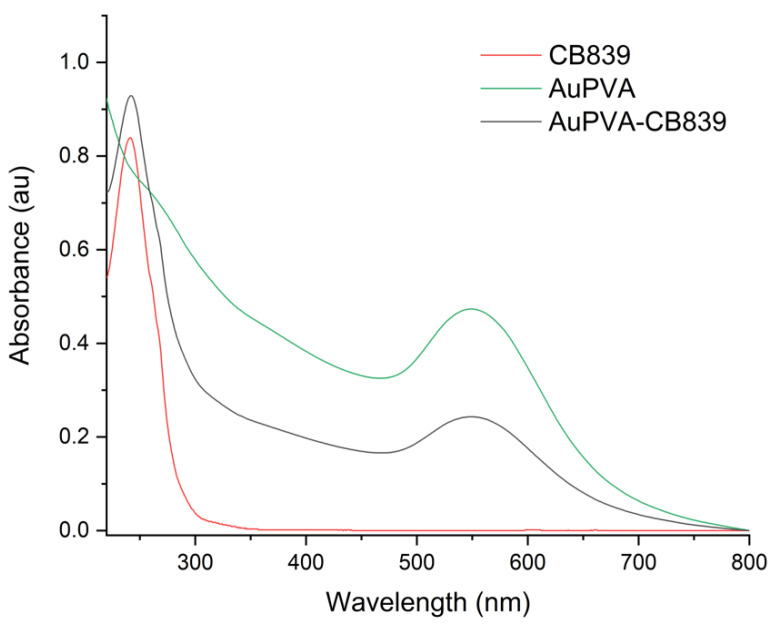
UV-VIS spectra of CB839 (red), AuPVA (green), and AuPVA-CB839 NPs (black).

Moreover, UV-VIS spectroscopy of the AuPVA-CB839 NPs showed an additional absorbance maximum at 240 nm corresponding to CB839 compared to the AuPVA NPs, which only showed the characteristic plasmonic band at 550 nm (Figure 4). These spectra also show no visible size increase or agglomeration of the AuPVA NPs after reaction with CB839, since the 550 nm absorbance signal of AuPVA-CB839 NPs did not shift to larger wavelengths.

**Figure 5:**
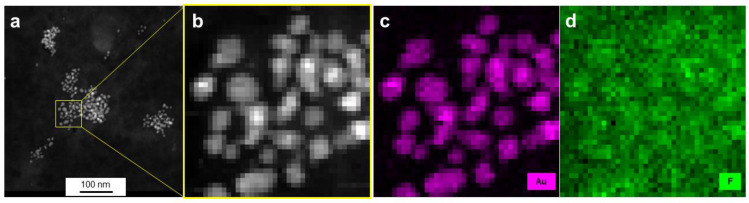
HAADF-STEM images (**a**,**b**) and EDX elemental mapping (**c**,**d**) of AuPVA-CB839 NPs. Gold, corresponding to the NPs is shown in pink and fluorine contained in CB839 in green.

Using energy-dispersive X-ray spectroscopy (EDX), elemental maps of AuPVA-CB839 NPs were collected on a scanning transmission electron microscope (STEM). The elements gold, corresponding to the AuPVA NPs and fluorine from CB839 are displayed in maps in Figure 5. Here, fluorine appears to be in the same area as gold and to form similar circular patterns correlated with the Au map. Since electrons scatter strongly from Au, the contrast in the fluorine (F-K) X-ray map is likely caused by an increase in the Bremsstrahlung background. In order to confirm the presence of F in the vicinity of Au NPs and to observe the influence of background radiation, X-ray spectra taken on Au NPs were compared against such taken from surrounding areas (Figure S7, SM).

From the Au-Mα_1_ elemental map (Figure S7a, SM), the inner areas and the edges of NPs were carefully selected (Figure S7b, SM) and three EDX spectra from these scan point subsets were created by averaging all points of a subset (Figure S7c, SM). Since the Au-Mα_1_ signal at 2123 eV (red dot, Figure S7c, SM) is strongest on the particles, weaker at edges and absent next to the particles, we believe the threshold values for the assignment to edge and particle interior were chosen correctly. There is no denying that stronger Bremsstrahlung occurs when the electron beam passes through the strongly scattering Au particles, as can be seen in the pure background regions of the spectra, e.g., at 1200–1500 eV. However, all spectra show a slight elevation of the EDX signal at 677 eV (F-K peak, black dot, Figure S7c, SM), independent of location. As AuPVA NPs are surrounded by a large-sized surface polymer able to bind CB839 molecules, a F-K emission around the NPs (black scan area on Figure S7b, SM) can also be expected. Thus, we conclude that even though the discovery of a low amount of F atoms on Au NPs present upon loading CB839 is difficult due to the detection limits of the equipment, CB839 is found near the Au NPs.

Thermogravimetric analysis (TGA, Figure S8, SM) showed that the AuPVA-CB839 conjugates are stable to about 200 °C.

The colloidal properties of AuPVA-CB839 NPs were also investigated using DLS measurements (Figure S9, SM). In water, the hydrodynamic diameter of AuPVA-CB839 NPs was approximately 20 nm, while in culture media (DMEM) their size increased to 80 nm. This larger NP size in DMEM is to be expected, due to the formation of a protein corona around the surface of NPs as they interact with several proteins contained in cell culture media [50].

### 3.4. In Vitro Effect of AuPVA-CB839 NPs in GSCs

Following these investigations, the first in vitro tests in classical cell models of adherently grown U87 cells were conducted with AuPVA, AuCit, and AuThioPEG NPs (Figure S10, SM). We followed in our analysis the previously recommended use of quantifying cell colony formation efficiency (CFE) to functionally assess the cytotoxicity of nanomaterials [41].

Because the ability of Au NPs to absorb visible light can lead to false positive results when using a photometer to determine cell viability (Figure S11, SM), a quantification with the CFE method is most suitable for our study. This assay avoids any interference of metals, in our case Au, with other test components or optical detection methods thus minimizing signal disturbance and false recordings. The biological assessment was performed in a robust in vitro platform of *n* = 4 different glioma models featuring *n* = 3 glioma stem cell systems. For each model, we quantified the ability to form monolayer and spheroidic colonies in the presence of different treatment conditions.

**Figure 6:**
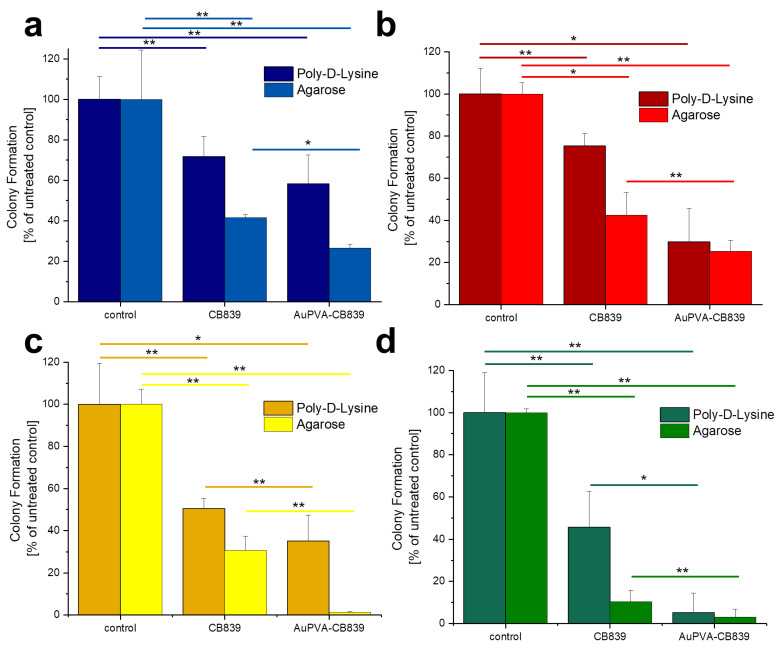
Colony Formation of U87 (**a**), JHH520 (**b**), GBM1 (**c**), and BTSC407 (**d**) cells after incubation with AuPVA-CB839 NPs, using both the 2D poly-d-lysine and the 3D agarose method. Dark columns in (**a**) are labelled as poly-d-lysine for comparison, even though no adhesion polymer was used since cell model U87 grows adherent already. A two-sample t-test was performed using Origin 8 software and significances are indicated: * *p* > 0.05, ** *p* > 0.03.

Given that JHH520, GBM1, and BTSC407 GSCs are not adherent, the CFE assay was modified using poly-d-lysine, a polycationic amino acid polymer, which is able to interact with anionic parts of neural cells, thus effectively attaching them to a surface. As Figure S12, SM shows, this attachment endured the NP and staining treatments of GBM cells and allowed for data interpretation by counting the colonies formed due to the ability of GSCs of making colonies from single cells [22]. The less colony formation, the more effective the drug treatment. Our tests reveal that treating the cells with AuPVA-CB839 NPs containing 1 µmol/L CB839 impaired tumor cell growth, as benchmarked to treatment with 1 µmol/L CB839 only (Figure 6). The differences in the amount of reduction on CFE between the 2D (poly-d-lysine) vs. 3D (agarose) assay could be explained by the different susceptibly of the cells when grown in different conditions, whereby 3D more closely recapitulated the actual tumor physiology [32]. In addition, the variations in experimental handling sequence could result in different error introduction into the assays (pretreatment of cells for setting the Agarose assay, washing steps in 2D poly-d-lysine assay). Importantly however, the general trend of augmented therapeutic potential of our nano-delivered CB839 as compared to the unmodified substance is shown in all tested cell models with both assays, thereby supporting our technology for the development of an anti-metabolic therapy potentially able to eradicate tumors with stem cell properties.

The percentage of colony formation in U87 cells with CB839 alone and with AuPVA-CB839 NPs differed only slightly when comparing both incubation methods (Figure 6a). JHH520 cells (Figure 6b) presented a similar reaction than GBM1 cells (Figure 6c), as they are both glutaminase-high expressing lines [22]. Interestingly, the in vitro clonogenicity of BTSC407 cells (Figure 6d) after treatment with AuPVA-CB839 NPs in agarose was only about 1%, compared to 30% with a CB839 treatment without NPs (relative to the control set at 100%), which speaks for the NP potential to enhance the drug effect, even in cell lines thought to be resistant due to lower GLS1 activation levels [22]. Based on our previously conducted functional assays with CB839 and other pharmacological GLS inhibitor C-968, using the same cell models as in this study, we believe that the total GLS1 protein abundancy predicts for the sensitivity of GBM cells to anti-GLS1 directed drugs. The fact that U87 and BTSC407 cells have comparably low GLS1 expression as opposed to GBM1 and JHH520 not only shows that our applied disease modeling technology can recapitulate the variation of target activity found in patients [21,22], but also reflects the molecular heterogeneous nature of this disease. Thus, this inter- and intratumoral cell metabolic heterogeneity may limit the durable effects of such monotherapies and the differences in saturation of GLS1 blockage could be a reason for the observed differences. Moreover, compared to serum-free spheroid models, the serum-containing in vitro growth conditions of the U87 cell line, known to dampen the effects of pharmaco interventions [51], can also be a reason for higher GLSi resistance. Acknowledging that this is rather a technical limitation than a translational relevant reason for possible source of therapy resistance, we added three spheroid, serum-free models (JHH520, BTSC407, and GBM1), in our project.

To our best knowledge, this is the first report of functionalizing the most promising GLSi compound CB839 to inorganic nanocarriers and performing bio validation. Our technology accompanies a previous report on BPTES-loaded poly(lactic)-glycolic acid NPs [52] and confirms the observation that application of nanotechnology is a powerful strategy to improve the therapeutic capacity of this drug class.

While the effect of CB839-loaded Au NPs on colony formation is shown, tests regarding quantification of the amount of inhibitor delivered into cells for each case are underway. Similarly, enzyme activity inhibition studies like those made in GSCs for CB839 [22] are pending for Au-CB839 NPs to gain additional knowledge about how they affect the target, as well as their intracellular delivery and release. Even though we recently benchmarked the phenotypic effects of CB839 against the absence of effects when applying DMSO in the same cell models as in the current study [22], we acknowledge that the repetition of these control experiments using ethanol solvent would be beneficial to further support some conclusions of this work. In the future, we will also include our CB839-loaded Au NPs in further screening assays in order to characterize their effect and toxicity more comprehensively in a high-throughput manner [53].

While the blood–brain barrier permeability of CB839 is challenging, as described in several in vitro studies [19,54], its specificity to inhibit GLS1 and the connection between response and IDH1 DNA mutation status of cancer patients [9] makes it a promising candidate for precision cancer therapy. Although an increased diffusion through the barrier is expected when attaching the small molecule to Au NPs [55,56,57], CB839 could also be part of an intraventrical, intrathecal, or localized intracranial chemotherapy. For intracranial chemotherapy, the BBB problematic could even be avoided because the drug is directly applied to the tumor cavity during tumor resection [58,59]. As has been well described in the literature for the study of GBM in vivo [60,61,62,63], we suggest a xenograft approach of human tumor stem cell models in rodent carriers, ideally in immune-tolerant models [64], to investigate the immune relevance of the applied intervention using nanocarried-CB839 in the future. Such an immunocompetent mouse model for human GBM cells—which has also been recently used for testing additional disease types [65]—would also help to study the effects of Au-CB839 NPs on the immune microenvironment of the tumor. Given the emergence of immune therapies as effective cancer treatments, the inclusion of such assays in future drug development projects are desirable to increase translational relevance.

### 3.5. Cell Internalization

**Figure 7:**
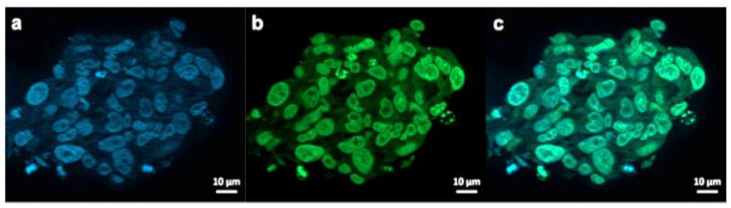
Exemplary fluorescence microscopy images of JHH520 cells after incubation with AuPVA-FITC NPs. Image (**a**) shows DAPI signals of cell DNA, image (**b**) FITC signals corresponding to AuPVA-FITC NPs and (**c**) a combined DAPI and FITC image.

To visualize the GSCs penetration potential by the Au-Polymer CB839 conjugate, fluorescent labelling of our NPs and our tumor models was used to investigate the NP internalization in GBM cells (Figure 7). For this purpose, AuPVA NPs were functionalized with FITC, resulting in AuPVA-FITC NPs. These fluorescent AuPVA NPs are used as a proof-of-concept to demonstrate that the PVA coated Au NPs are able to penetrate GSCs. We have only fluorescent-stained the AuPVA NPs with FITC because these NPs had the highest DLE. From our previous work [25], we realized the importance of the surface ligand on the degree of NP internalization and had discovered differences when testing the same NPs in different GBM cell lines. Thus, we wanted to make sure that AuPVA NPs were able to penetrate GSCs if they are later to be loaded with CB839.

The successful attachment of FITC to the Au NPs relies on the presence of a sulfur atom in the isothiocyanate group of the fluorophore. Given the strong affinity of gold for sulfur, FITC is able to coordinatively bind to Au NPs though relatively stable bonds [66,67]. Free FITC with its isothiocyanato, -NCS group is able to form Au-S bonds on the gold metal surface, requiring only minimal reaction time [68,69]. This attachment was confirmed by fluorescence spectroscopy (Figure S13, SM), which showed a quenched but still present fluorescence signal of AuPVA-FITC compared to FITC due to the interaction of the fluorophore with Au [70]. Moreover, the stretching band of the C=O group of the lactone in FITC was visible at 1728 cm^−1^ in IR spectra taken from AuPVA-FITC NPs (Figure S14, SM), as well as stretching vibrations from the benzene ring at 1601 and 1435 cm^−1^. The FITC loading efficiency of AuPVA-FITC NPs (see Equation (S1) in the SM) was calculated using UV-VIS spectroscopy (Figure S15, SM). By comparing the absorbance intensity of the supernatant recovered after NP centrifugation (unloaded FITC) and the absorbance of total FITC used for the reaction, the loading was estimated to be approximately 23%. According to zeta potential measurements, AuPVA-FITC NPs remain stable in water for at least one hour at room temperature (Figure S16, SM). After this time, the surface charge of the NPs (−16 mV at 0 min) continues to decrease until reaching a zeta value of −27 mV, similar to the value of AuPVA NPs without FITC. Therefore, only freshly-prepared AuPVA-FITC NPs were used for further experiments in order to guarantee that a sufficient amount of FITC was still present on the NPs.

AuPVA-FITC NPs were added to the cells and sphere sections were studied under a fluorescence microscope. After staining cell DNA (Figure 7a), a green FITC signal (Figure 7b) corresponding to the NPs were visible in the same area (Figure 7c). Thus, we conclude that PVA is a suitable capping ligand for Au NPs, as it also allows their internalization, which is visualized after quenching extracellular fluorescence using Trypan Blue (Figure S17, SM).

Even though it has been demonstrated that Au NPs can be internalized through various endocytosis-based pathways or by direct cytoplasmic uptake [71], additional experiments are needed to determine the internalization mechanism of Au-CB839 NPs.

## 4. Conclusions

The synthesis of five Au NP types (four with polymer ligands and one with citrate) resulted in particles of 8–15 nm in size with different polymeric capping ligands. Despite the lack of functional groups with high chemical reactivity and a significant steric hindrance from its large size, the CB839 molecule could be immobilized on the surface of Au NPs through physical adsorption and electrostatic interactions with the polymers. AuPVA NPs were able to immobilize the highest amount of CB839, displaying a DLE of 12%. Calculations estimated that at least 60% of all AuPVA NPs carry one CB839 molecule. Even though characterization and quantification of CB838 loading using chromatographic and spectroscopic methods is challenging, AuPVA-CB839 NPs demonstrated improved biological results. In vitro experiments in GSCs showed an enhanced effect of CB839 when delivered by Au NPs as compared to the neat drug solution. As expected due to the cellular and molecular heterogeneity of GBM, the therapy effect varied depending on the cell lines used. Our study presents and compares two suitable bioassays to test the effect of CB839-loaded Au nanocarriers. While 3D cell cultures are better models to mimic in vivo conditions, 2D cultures greatly facilitate a treatment with nanomaterials. Although human cell assays emerge as the preferred option to assess therapy efficacy and toxicology risk, current Au-drug development projects require testing in humanized animal models to enhance clinical prediction. Given the previously unmet need of treating GBM with effective, long-lasting therapies, our work raises hope to improve future care of patients using anti-cell metabolism directed approaches. Moreover, exploiting the unique optical and magnetic properties of Au NPs as theranostic devices are underway, thereby further extending the list of options how inorganic chemistry has the potential to improve neuro-oncology care, including clinical applied strategies such as photo- or hyperthermal therapy.

## Figures and Tables

**Table 1:** Drug loading efficiency of Au NPs ^a^.

Au NPs	Drug Loading Efficiency (%)
AuCit	8
AuThioPEG	4
AuPVA	12
AuPVP	0
AuPEI	1

^a^ From HPLC determination in supernatant solution according to Equation (1).

## Data Availability

The data presented in this study is contained within this article and its supplementary materials.

## Supplementary Materials

The following are available online at https://www.mdpi.com/1999-4923/13/2/295/s1, Figure S1: Structural formula of citric acid (H_3_Cit) and of the repeat units of the polymers poly(ethylene glycol) methyl ether thiol (ThioPEG), polyvinyl alcohol (PVA), polyvinyl pyrrolidone (PVP) and branched polyethylene imine (PEI), Figure S2: Histograms of Au-citrate (AuCit) (a), AuThioPEG (b), AuPVA (c) and AuPVP (d) nanoparticles (NPs). ThioPEG = poly(ethylene glycol) methyl ether thiol, PVA = polyvinyl alcohol, PVP = polyvinyl pyrrolidone., Figure S3: TEM images and histograms of AuCit-CB839 (a,f), AuThioPEG-CB839 (b,g), AuPVA-CB839 (c,h), AuPVP-CB839 (d,i) and AuPEI-CB839 (e,j) NPs. Images were captured at 200,000× (a), 100,000× (b) and 150,000× (c,d,e) magnification, Figure S4: HPLC chromatogram (a) and standard calibration curve (b) used for CB839 quantification., Figure S5: Structures of the two CB839 molecules, extracted from “glutaminase C in complex with inhibitor CB-839” (PBD ID: 5HL1, MMDB ID: 142270) [1]. Hydrogen atoms were added with Mercury [2], Figure S6: IR spectra of AuPVA-CB839 NPs (in black), CB839 (in red) and AuPVA NPs (in green), Figure S7: STEM EDX analysis of AuPVA-CB839 NPs. Elemental maps from the Au-Mα_1_ intensity at 2123 ± 60 eV (a), NP inner and edge area selection ((b), all scan points assigned to edges are red, all those assigned to NP interiors are yellow and all other points are black) and corresponding EDX spectra of the three areas (c). Note: In (a) image pixels represent points where the electron probe was placed. A scan of 40 × 40 pixels was performed so, Figure S7a is shown at raw data resolution, Figure S7b is a mask applied to spectroscopic images at the resolution of the measurement, Figure S8: TGA curve for the decomposition of AuPVA-CB839 NPs (in black), CB839 (in red) and PVA (in blue), Figure S9: DLS measurements of AuPVA-CB839 NPs in water(a) and in DMEM (b), Figure S10: Colony Formation after treatment of U87 cells with CB839, AuThioPEG-CB839, AuCit-CB839 and AuPVA-CB839 NPs. Untreated cells were used as control, Figure S11: Absorbance measurements after incubation of GBM1 cells with culture medium (Control), AuPVA and AuPVA-CB839 NPs. These values were measured after performing the MTT Assay, as described previously [3]. Even though the MTT absorbance should be highest at the control (highest possible cell viability under the chosen conditions), wells treated with Au NPs show superior absorbance values even after several washing steps to remove NPs. This observation stresses the importance of choosing label-free non-optical methods to assess the toxicity of compounds accompanied by metal NPs, Figure S12: BTSC407 colonies after incubation with CB839 (wells 1–3) and with AuPVA-CB839 NPs (wells 4–6) using poly-d-lysine, Figure S13: Fluorescence spectra of AuPVA-FITC NPs (red line) and FITC (green line), Figure S14: IR spectra of AuPVA-FITC NPs (in brown), FITC (in purple) and AuPVA NPs (in green), Figure S15: UV-VIS spectra used to determine the loading efficiency of FITC for AuPVA-FITC NPs. The purple (upper) line (FITC) indicates the absorbance of total FITC added to the reaction. The brown (lower) line (sup. AuPVA-FITC) corresponds to the absorbance of FITC, which was not loaded onto AuPVA NPs and was obtained from the supernatant after centrifugation of the AuPVA-FITC product, Figure S16: Zeta potential measurements of AuPVA-FITC NPs. (a): Comparison of zeta potential of AuPVA NPs (in red) and freshly-prepared AuPVA-FITC NPs (in green). (b): Time-dependent zeta potential values of AuPVA-FITC NPs in water at room temperature, Figure S17: Microscopic images of JHH520 cells with AuPVA-FITC NPs after Trypan Blue quenching of extracellular fluorescence. (a) shows DAPI staining of cell nuclei, (b) shows the fluorescent AuPVA-FITC NPs inside the cells and (c) is a combination of (a,b).
