# Supplementary material for: Augmented Therapeutic Potential of Glutaminase Inhibitor CB839 in Glioblastoma Stem Cells Using Gold Nanoparticle Delivery
Source: Pharmaceutics. 2021 Feb 23;13(2):295. doi: 10.3390/pharmaceutics13020295 (PMC7926460; doi:10.3390/pharmaceutics13020295)
Supplement: Supplementary file 1 [file pharmaceutics-13-00295-s001.pdf]

pharmaceutics

MDPI

Supplementary Materials: Augmented Therapeutic Potential of Glutaminase Inhibitor CB839 in Glioblastoma Stem Cells Using Gold Nanoparticle Delivery

Beatriz Giesen, Ann-Christin Nickel, Juri Barthel, Ulf Dietrich Kahlert and Christoph Janiak

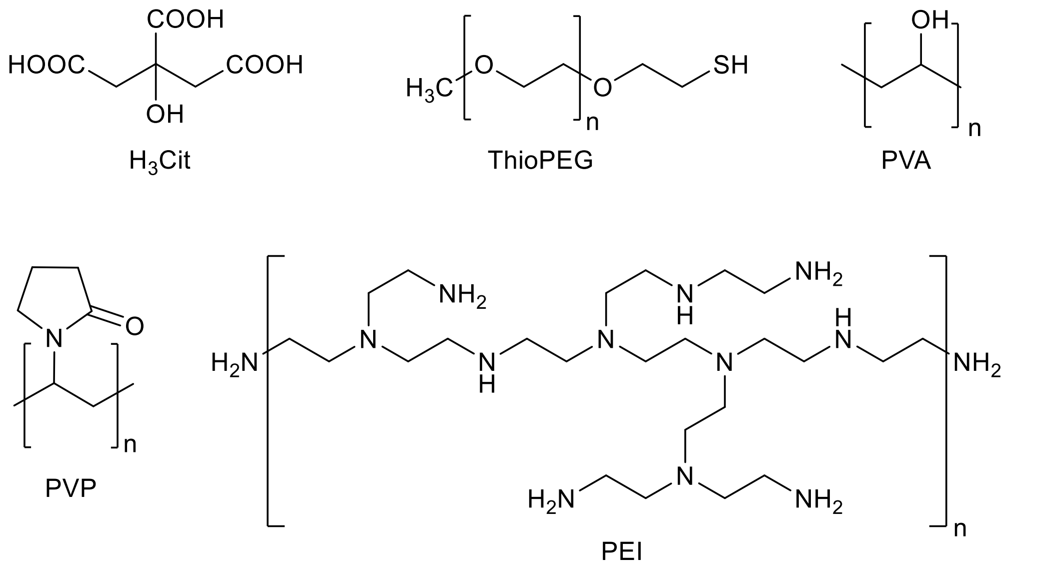

Figure S1. Structural formula of citric acid (H_3Cit) and of the repeat units of the polymers poly(ethylene glycol) methyl ether thiol (ThioPEG), polyvinyl alcohol (PVA), polyvinyl pyrrolidone (PVP) and branched polyethylene imine (PEI).

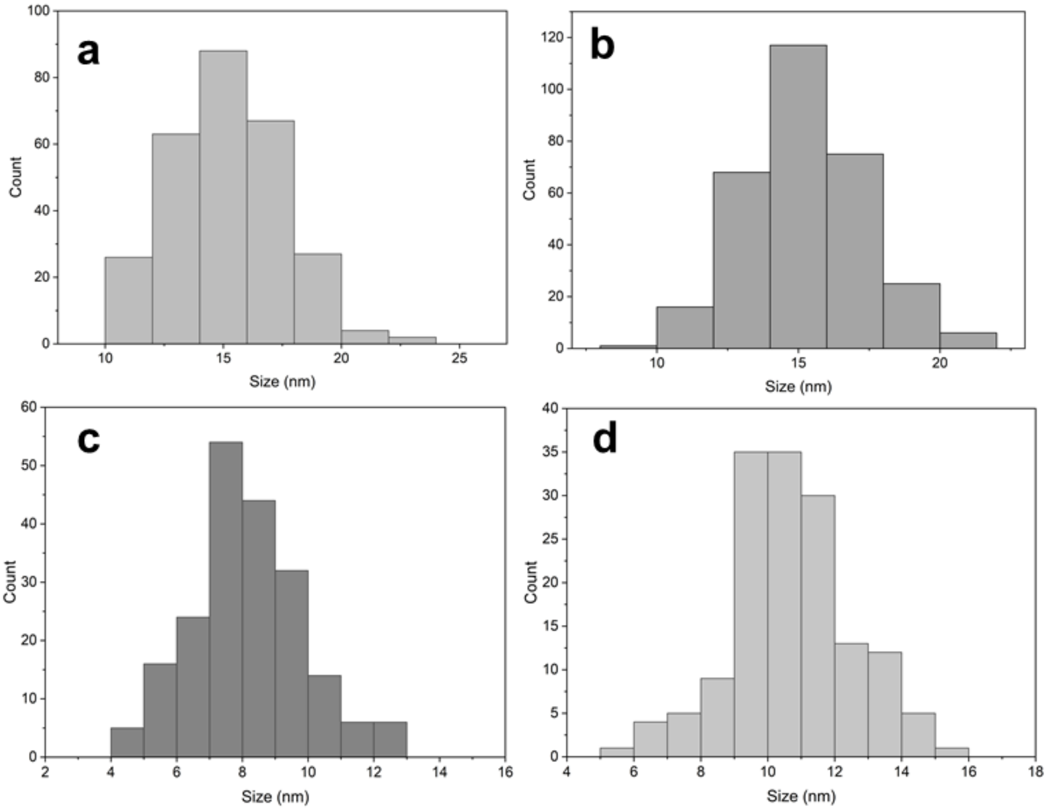

Figure S2. Histograms of Au-citrate (AuCit) (**a**), AuThioPEG (**b**), AuPVA (**c**) and AuPVP (**d**) nanoparticles (NPs). ThioPEG = poly(ethylene glycol) methyl ether thiol, PVA = polyvinyl alcohol, PVP = polyvinyl pyrrolidone.

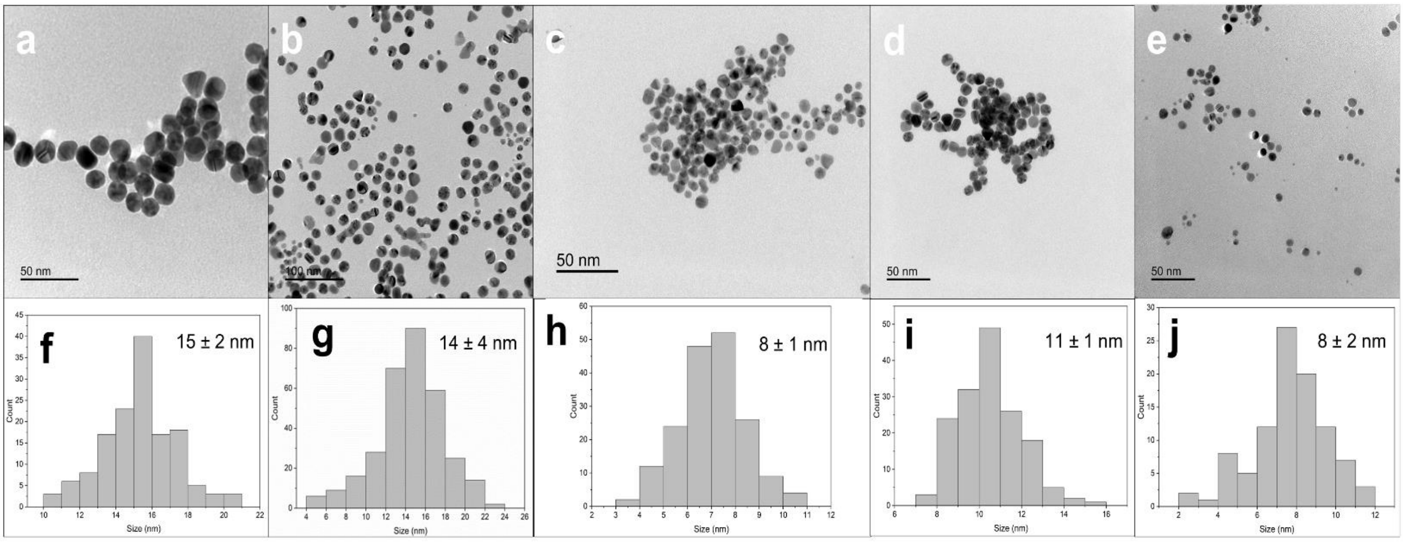

Figure S3. TEM images and histograms of AuCit-CB839 (**a**,**f**), AuThioPEG-CB839 (**b**,**g**), AuPVA-CB839 (**c**,**h**), AuPVP-CB839 (**d**,**i**) and AuPEI-CB839 (**e**,**j**) NPs. Images were captured at 200,000× (**a**), 100,000× (**b**) and 150,000× (**c**,**d**,**e**) magnification.

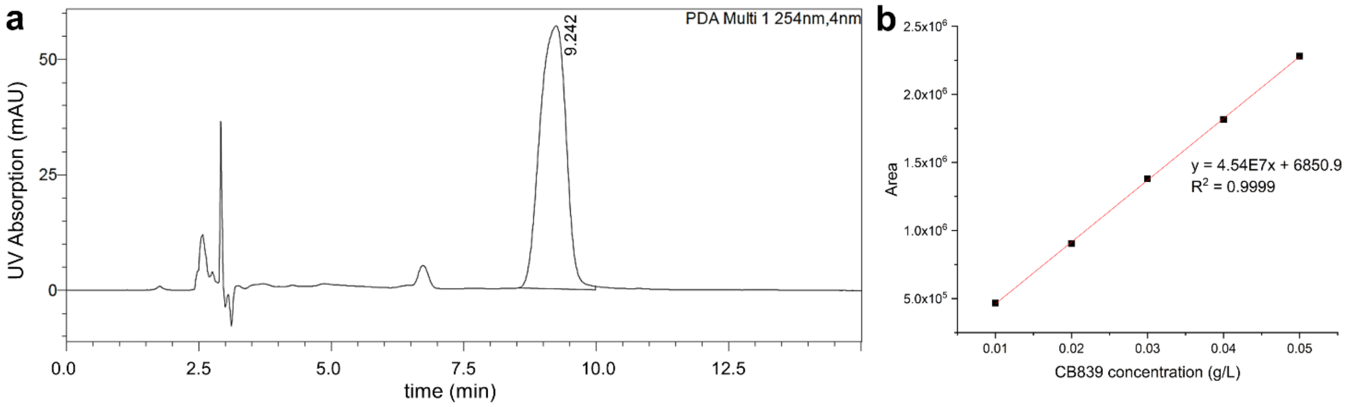

Figure S4. HPLC chromatogram (**a**) and standard calibration curve (**b**) used for CB839 quantification.

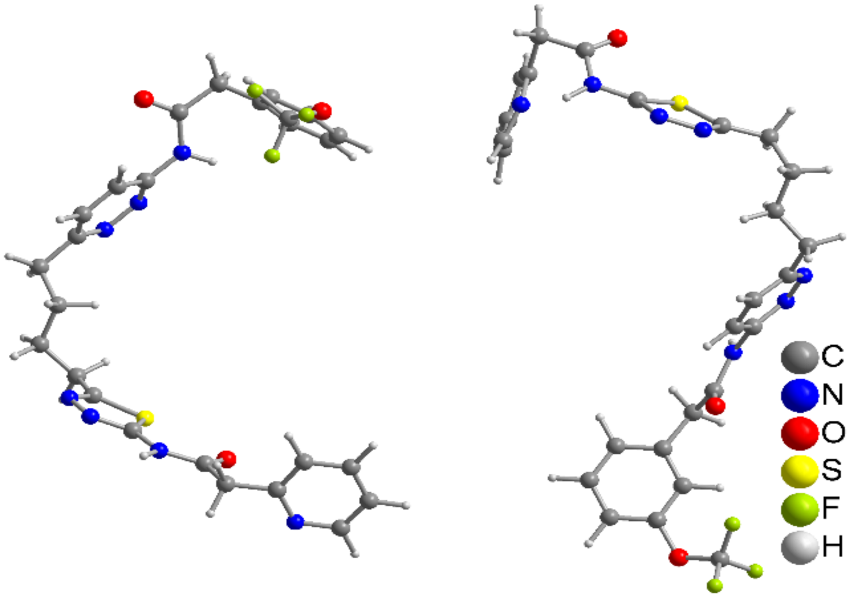

Figure S5. Structures of the two CB839 molecules, extracted from "glutaminase C in complex with inhibitor CB-839" (PBD ID: 5HL1, MMDB ID: 142270) [1]. Hydrogen atoms were added with Mercury [2].

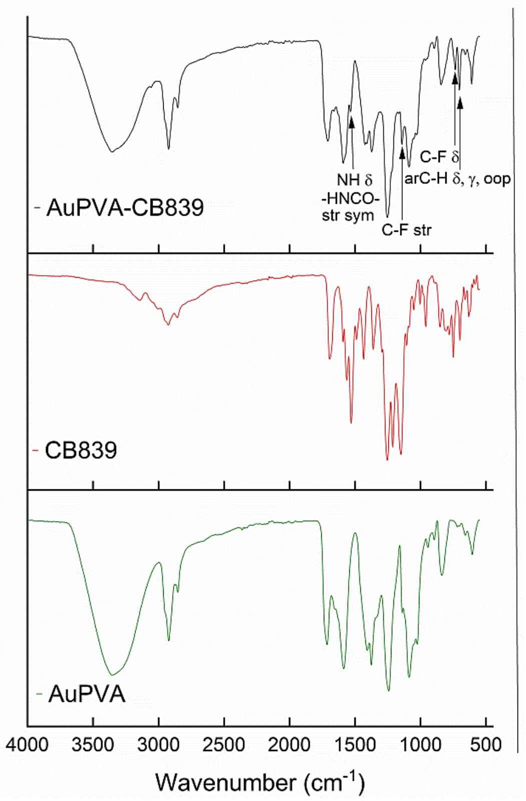

Figure S6. IR spectra of AuPVA-CB839 NPs (in black), CB839 (in red) and AuPVA NPs (in green).

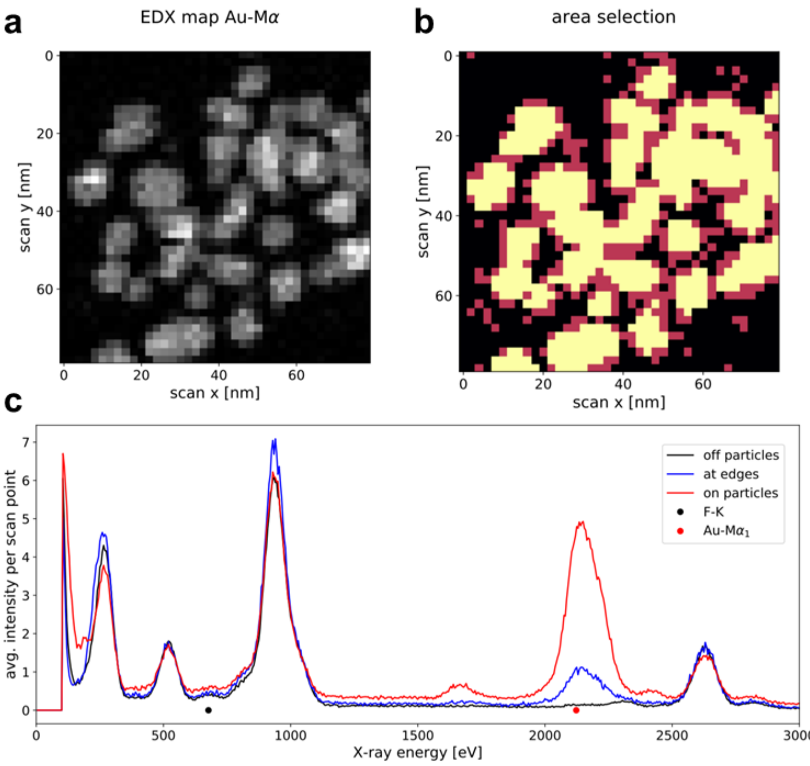

Figure S7. STEM EDX analysis of AuPVA-CB839 NPs. Elemental maps from the Au-Mα1 intensity at 2123 ± 60 eV (**a**), NP inner and edge area selection ((**b**), all scan points assigned to edges are red, all those assigned to NP interiors are yellow and all other points are black) and corresponding EDX spectra of the three areas (**c**). Note: In (a) image pixels represent points where the electron probe was placed. A scan of 40 × 40 pixels was performed so, Figure S7a is shown at raw data resolution, Figure S7b is a mask applied to spectroscopic images at the resolution of the measurement.

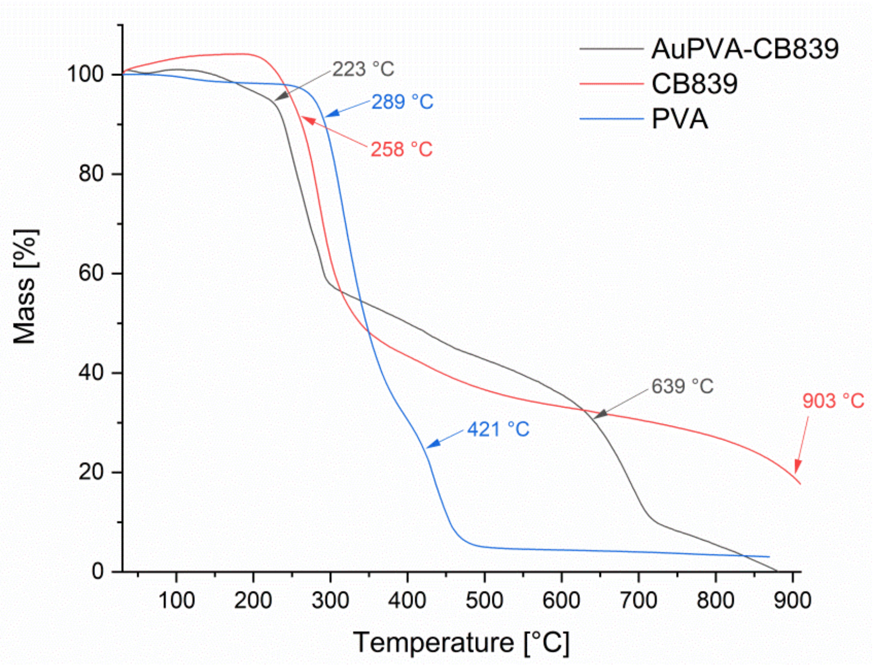

Figure S8. TGA curve for the decomposition of AuPVA-CB839 NPs (in black), CB839 (in red) and PVA (in blue).

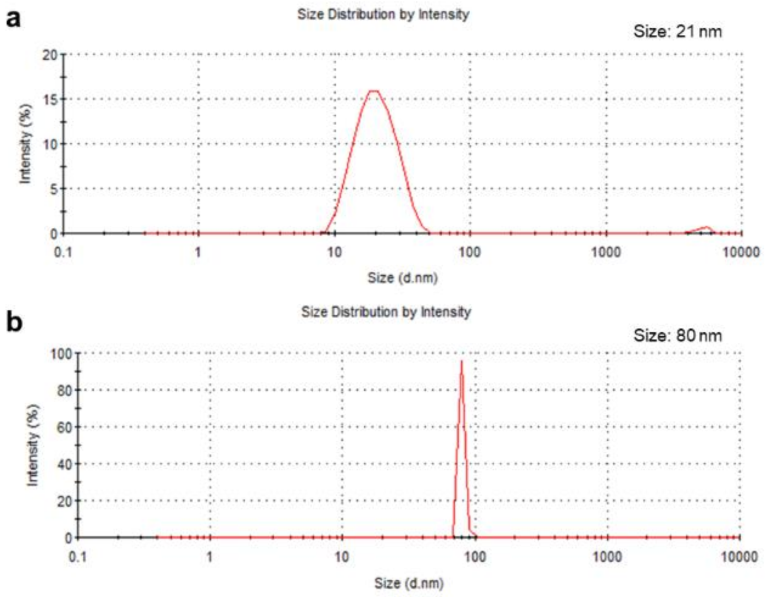

Figure S9. DLS measurements of AuPVA-CB839 NPs in water(**a**) and in DMEM (**b**).

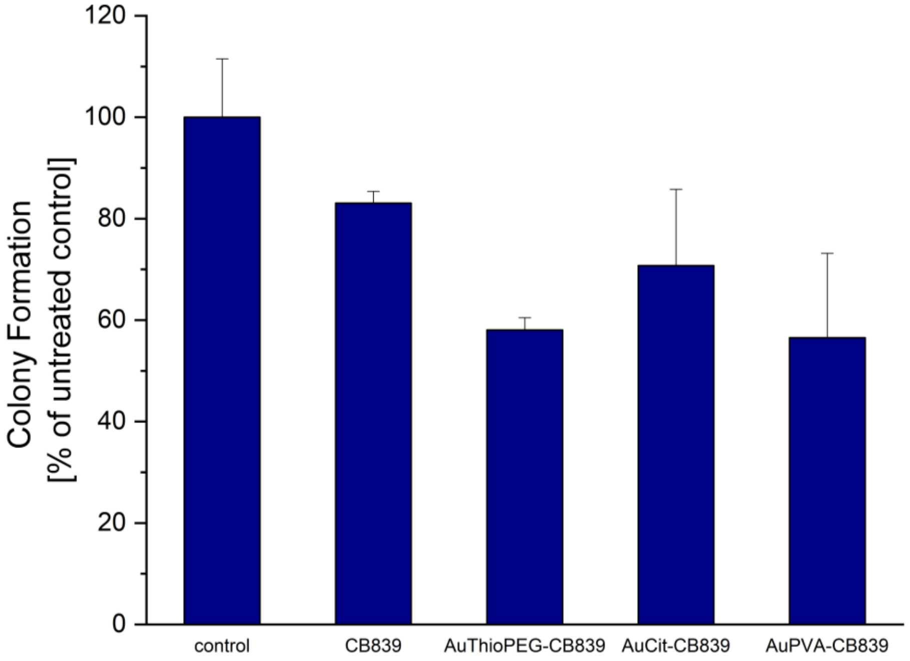

Figure S10. Colony Formation after treatment of U87 cells with CB839, AuThioPEG-CB839, AuCit-CB839 and AuPVA-CB839 NPs. Untreated cells were used as control.

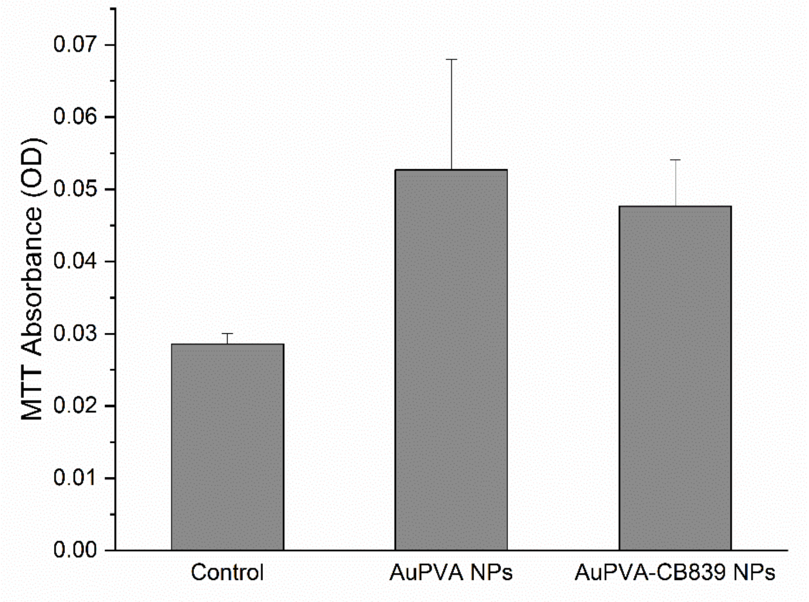

Figure S11. Absorbance measurements after incubation of GBM1 cells with culture medium (Control), AuPVA and AuPVA-CB839 NPs. These values were measured after performing the MTT Assay, as described previously [3]. Even though the MTT absorbance should be highest at the control (highest possible cell viability under the chosen conditions), wells treated with Au NPs show superior absorbance values even after several washing steps to remove NPs. This observation stresses the importance of choosing label-free non-optical methods to assess the toxicity of compounds accompanied by metal NPs.

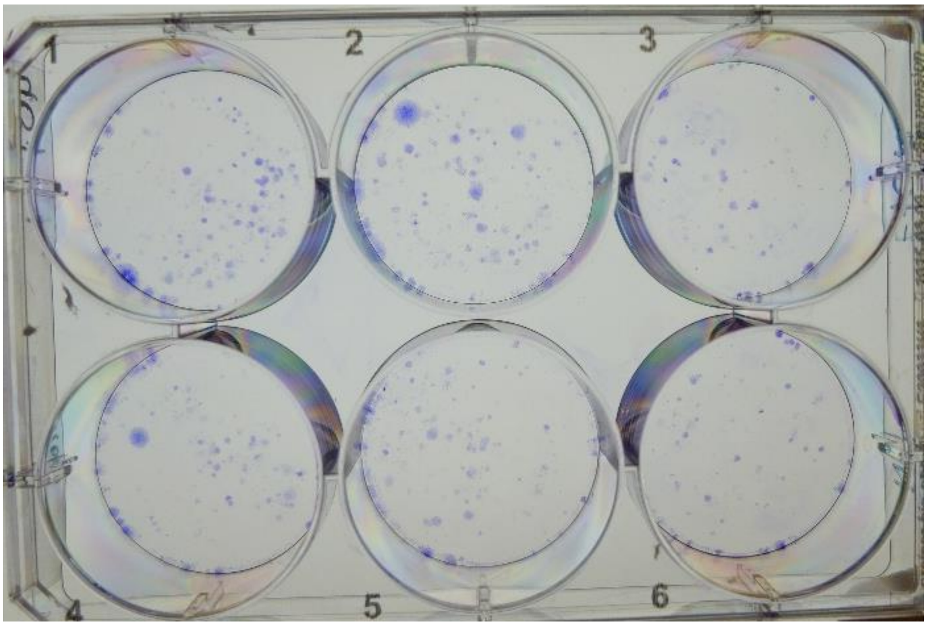

Figure S12. BTSC407 colonies after incubation with CB839 (wells 1–3) and with AuPVA-CB839 NPs (wells 4–6) using poly-D-lysine.

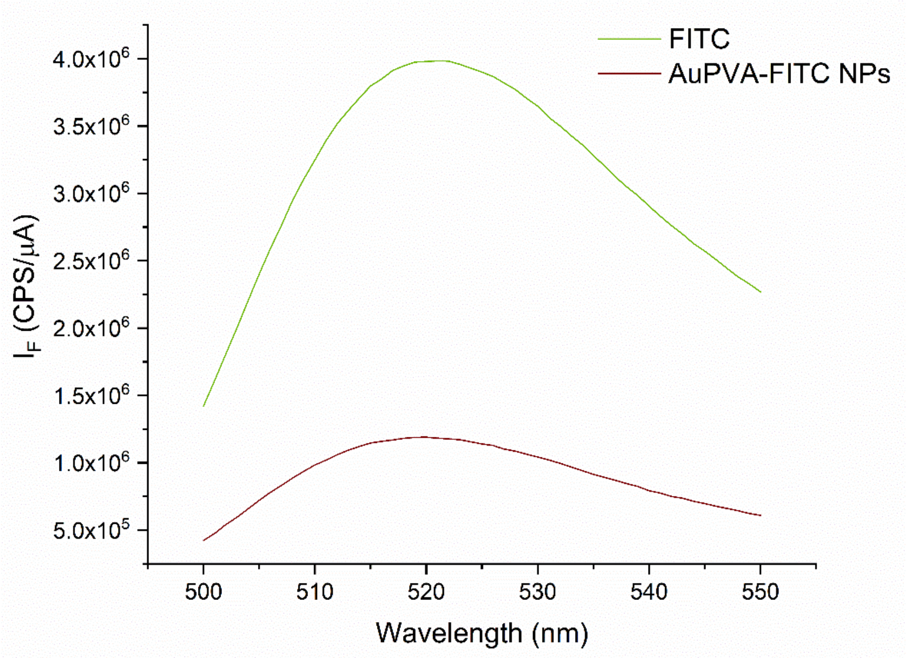

Figure S13. Fluorescence spectra of AuPVA-FITC NPs (red line) and FITC (green line).

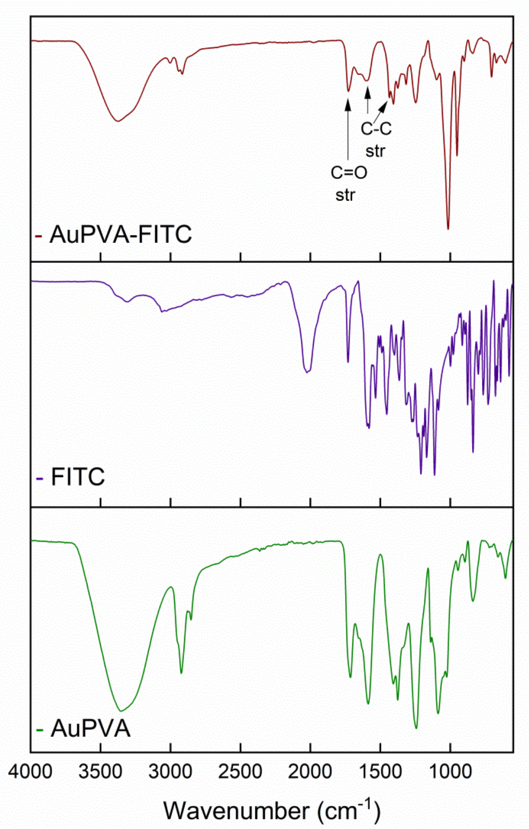

Figure S14. IR spectra of AuPVA-FITC NPs (in brown), FITC (in purple) and AuPVA NPs (in green).

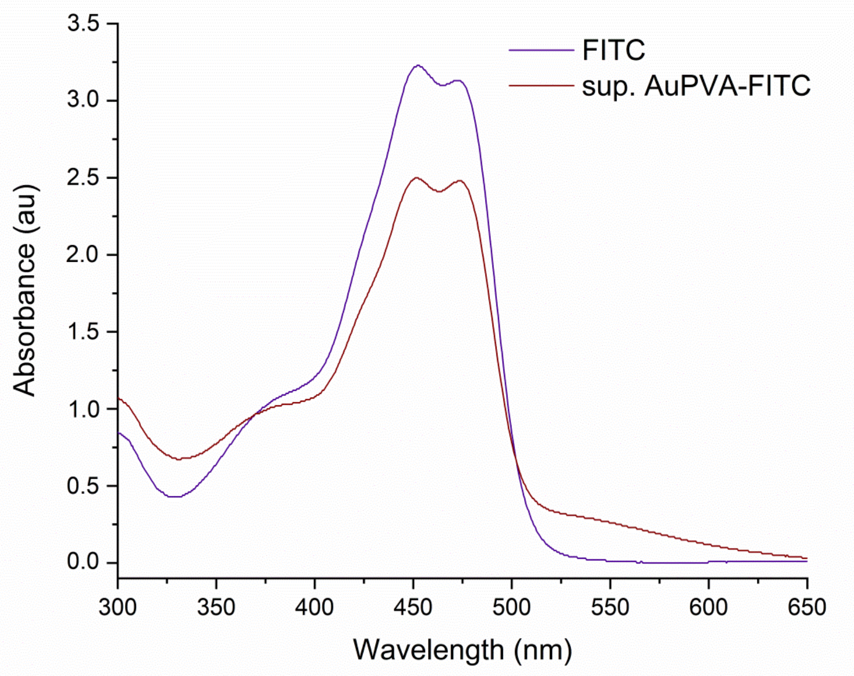

Figure S15. UV-VIS spectra used to determine the loading efficiency of FITC for AuPVA-FITC NPs. The purple (upper) line (FITC) indicates the absorbance of total FITC added to the reaction. The brown (lower) line (sup. AuPVA-FITC) corresponds to the absorbance of FITC, which was not loaded onto AuPVA NPs and was obtained from the supernatant after centrifugation of the AuPVA-FITC product.

$$FITC\ Loading\ Efficiency\ (\%) = \frac{Absorbance\ FITC\ (450\ nm) - \text{absorbance sup. AuPVA} - \text{FITC (450 nm)}}{Absorbance\ FITC\ (450\ nm)} \times 100\%$$

$$= \frac{3.23 - 2.5}{3.23} \times 100\% = 23\% \quad \text{(S1)}$$

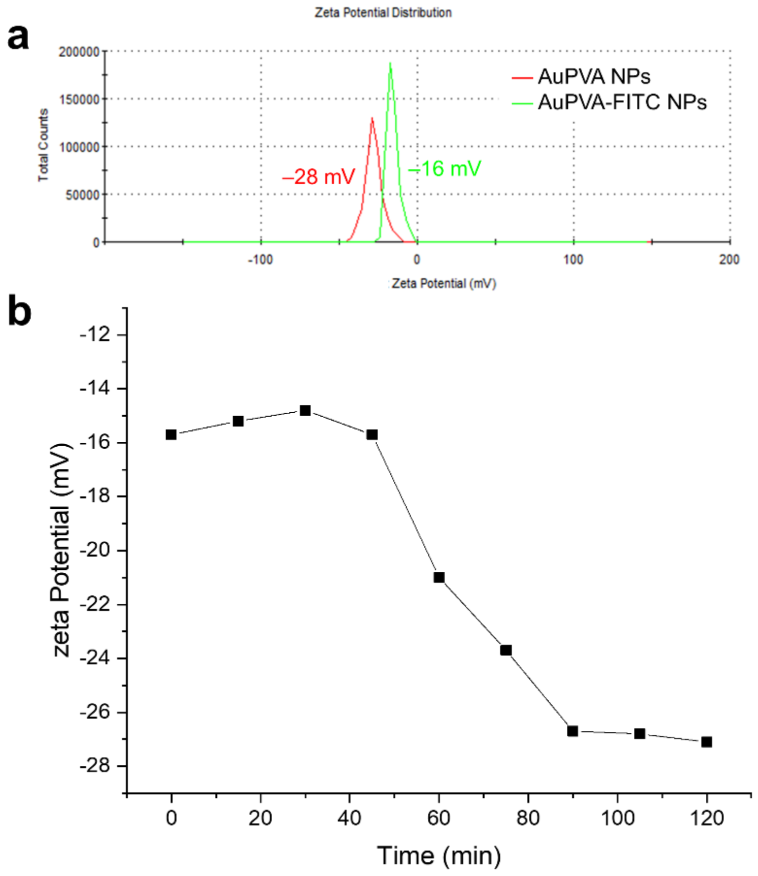

Figure S16. Zeta potential measurements of AuPVA-FITC NPs. (**a**): Comparison of zeta potential of AuPVA NPs (in red) and freshly-prepared AuPVA-FITC NPs (in green). (**b**): Time-dependent zeta potential values of AuPVA-FITC NPs in water at room temperature.

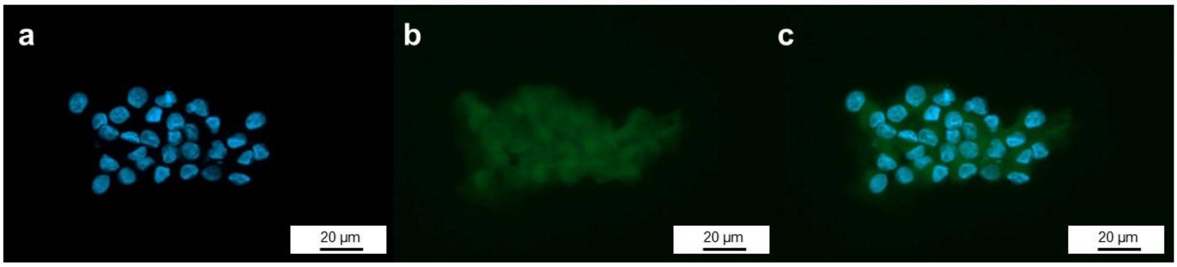

Figure S17. Microscopic images of JHH520 cells with AuPVA-FITC NPs after Trypan Blue quenching of extracellular fluorescence. (**a**) shows DAPI staining of cell nuclei, (**b**) shows the fluorescent AuPVA-FITC NPs inside the cells and (**c**) is a combination of (**a**) and (**b**).

References

1. Huang, Q., Stalnecker, C., Zhang, C., McDermott, L. A., Iyer, P., O'Neill, J., Reimer, S., Cerione, R. A. and Katt, W. P. Characterization of the interactions of potent allosteric inhibitors with glutaminase C, a key enzyme in cancer cell glutamine metabolism. *J. Biol. Chem.* **2018**, 293, 3535–3545.
2. Mercury 2020.2.0, Copyright © CCDC, 2001–2020, http://www.ccdc.cam.ac.uk/
3. Giesen, B., Nickel, A.-C., Garzón-Manjón, A., Vargas-Toscano, A., Scheu, C., Kahlert, U. D. and Janiak, C. Influence of synthesis methods on the internalization of fluorescent gold nanoparticles into glioblastoma stem-like cells. *J. Inorg. Biochem.* **2020**, 203, 110952.
